# Modulation of the Activity of *Mycobacterium tuberculosis* LipY by Its PE Domain

**DOI:** 10.1371/journal.pone.0135447

**Published:** 2015-08-13

**Authors:** Christopher K. Garrett, Lindsey J. Broadwell, Cassandra K. Hayne, Saskia B. Neher

**Affiliations:** 1 Department of Biochemistry and Biophysics, University of North Carolina at Chapel Hill, Chapel Hill, NC, United States of America; 2 Department of Chemistry, University of North Carolina at Chapel Hill, Chapel Hill, NC, United States of America; The Catholic University of the Sacred Heart, Rome, ITALY

## Abstract

*Mycobacterium tuberculosis* harbors over 160 genes encoding PE/PPE proteins, several of which have roles in the pathogen’s virulence. A number of PE/PPE proteins are secreted via Type VII secretion systems known as the ESX secretion systems. One PE protein, LipY, has a triglyceride lipase domain in addition to its PE domain. LipY can regulate intracellular triglyceride levels and is also exported to the cell wall by one of the ESX family members, ESX-5. Upon export, LipY’s PE domain is removed by proteolytic cleavage. Studies using cells and crude extracts suggest that LipY’s PE domain not only directs its secretion by ESX-5, but also functions to inhibit its enzymatic activity. Here, we attempt to further elucidate the role of LipY’s PE domain in the regulation of its enzymatic activity. First, we established an improved purification method for several LipY variants using detergent micelles. We then used enzymatic assays to confirm that the PE domain down-regulates LipY activity. The PE domain must be attached to LipY in order to effectively inhibit it. Finally, we determined that full length LipY and the mature lipase lacking the PE domain (LipYΔPE) have similar melting temperatures. Based on our improved purification strategy and activity-based approach, we concluded that LipY’s PE domain down-regulates its enzymatic activity but does not impact the thermal stability of the enzyme.

## Introduction

*Mycobacterium tuberculosis (Mtb)* is remarkably adept at interfering with host cellular processes in order to evade destruction. This ability depends on the secretion of virulence factors that modify the host environment. One family of proteins, known as PE and PPE proteins, are involved in immune evasion and virulence[1–3]. PE/PPE proteins are unique to mycobacteria; they were initially discovered when sequencing of the *Mtb* genome revealed approximately 160 genes encoding proteins with Pro–Glu (PE) or Pro–Pro–Glu (PPE) motifs near their N-termini[4]. Subsequent analysis revealed that PE/PPE proteins comprise about 7% of the coding capacity of the *Mtb* genome[5]. Although PE/PPE domains have been identified in both pathogenic and saprophytic mycobacteria, pathogenic mycobacteria maintain the highest number of PE/PPE proteins[6].

The PE motif is a moderately conserved, 110-residue domain found at the N-terminus of PE proteins[4]. The PPE motif is a distinct, but also conserved, domain of about 180 residues found at the N-terminus of PPE proteins[4]. The C-terminal domains of both PE and PPE proteins are highly variable and can encode enzymatic domains, conserved sequence motifs or large, repeated arrays of peptide motifs[4, 5]. Genes encoding PE and PPE proteins are often proximal on the *Mtb* genome and functionally linked[7]. In fact, structural studies show that in some cases, PE and PPE proteins form heterodimeric complexes[8]. PE/PPE gene families co-evolved with specialized, type VII secretion systems important to *Mtb* virulence known as the ESX secretion systems[9]. The *Mtb* genome encodes five type VII secretion systems, named ESX-1 to ESX-5[10]. Studies using both *Mtb* and *Mycobacterium marinum* revealed that several PE and PPE proteins depend on ESX-5 for export[11, 12].

LipY is a PE protein with a C-terminal triglyceride (TG) lipase domain[13]. LipY is proposed to have a dual role in *Mtb* pathogenesis[14]. First, *Mtb* is known to store host-derived TGs in lipid droplets that provide fuel during reactivation from dormancy[15–17]. LipY is the primary contributor to the break down of these stored TGs[13]. Next, overexpression of LipY has been implicated in increased *Mtb* virulence as shown by the enhanced mortality of TB-infected mice[14]. The increased mortality associated with LipY overproduction is attributed to down-regulation of host immunity by the products of LipY TG hydrolysis[14, 18]. These two roles for LipY are consistent with the observation that LipY is found both intracellularly and on the cell exterior[19].

LipY lacks a classic secretion signal but contains an YxxxD/E motif (Y-A-A-A-E) beginning at position 88 of its PE domain. The YxxxD/E motif is found in several other PE proteins and appears to be a general secretion signal required for recognition by the ESX-1 and ESX-5 secretion systems[20]. In LipY the motif is essential for secretion by ESX-5[20]. In some ESX and PE/PPE protein pairs, the YxxxD/E motif in one protein forms a joint motif with the sequence WxG present in its partner[21]. However, there is little evidence to suggest LipY has a PPE binding partner necessary for secretion[8, 22].

Upon export to the cell wall, LipY’s PE domain is removed by proteolytic cleavage[19]. One study using the cell wall fraction of *Mycobacterium smegmatis* containing LipY hinted that LipY’s PE domain could down-regulate its enzymatic activity[23]. This study also showed that mycobacteria expressing LipY lacking its PE domain exhibited a greater reduction in intracellular TG pools than mycobacteria expressing LipY. Therefore, it appears that although the Y-A-A-A-E motif in the N-terminus of LipY is necessary for its export to the cell wall, the PE domain likely has additional, unexplored functions. Here, we utilize biochemical assays with purified proteins and determined that LipY’s PE domain regulates its enzymatic activity.

## Materials and Methods

### LipY, LipYΔPE and PE Domain Purification

LipY, LipYΔPE (amino acids 150–437) and the PE Domain (1–149) were cloned into pET16b expression vectors (Novagen) with a C-terminal His tag. The proteins were expressed in BL21 (DE3) *E*. *coli* cells and grown to an OD_600_ of ~0.7 at 37°C. Protein expression was induced with 0.7 mM isopropyl-beta-D-thiogalactopyranoside and cells were shaken at 18°C for 16 h. Cells were then pelleted at 5,900 x g for 25 min and the pellet suspended in buffer A (20 mM HEPES pH 7, 300 mM NaCl, 10 mM imidazole, 10% glycerol) before being lysed in a Nano DeBEE Laboratory homogenizer (BEE International) at 20,000 psi. The lysates were cleared by centrifugation at 23,000 x g for 40 minutes. The resulting pellet was resuspended in buffer B (20 mM HEPES pH 7, 300 mM NaCl, 10 mM imdazole, 10% glycerol, 0.5% w/v N-laurylsarcosine) with agitation at 4°C for 1 hour (incubation time is critical) followed by centrifugation at 23,000 x g for 40 minutes. The supernatant was then incubated with pre-equilibrated nickel-nitrilotriacetic acid resin (Qiagen) with gentle agitation for 1 hour. The resin was washed with 4 column volumes of buffer A followed by elution with buffer C (20 mM HEPES pH 7, 300 mM NaCl, 350 mM imidazole, 10% glycerol). Eluted protein was incubated with Tween 20 with gentle agitation for 1 hour at 4°C and then loaded onto a pre-equilibrated Superdex 200 10/300 HR gel filtration column (GE Healthcare) with Buffer D (20 mM HEPES pH 7, 100 mM NaCl, 10% glycerol). Protein was eluted over 1 column volume with Buffer D. Protein that eluted between 8 and 10 mLs was collected and labeled ‘Peak 1’ while protein that eluted between 12 and 14 mL was labeled ‘Peak 2’. Protein was flash frozen in liquid nitrogen and stored at -80°C. A final purity of >95% was achieved for both LipY and LipYΔPE (S1 Fig). Because LipY and LipYΔPE were purified with detergent, we tested their ability to hydrolyze a natural triglyceride substrate in buffer that did not contain detergent and found that both were active (S2 Fig).

### Quantification of Active LipY and LipYΔPE by Activity-Based Protein Profiling (ABPP)

LipY and LipYΔPE were diluted to the same concentration using ABPP buffer (20 mM Tris pH 8, 100 mM NaCl). A 1.5 molar excess of ActivX TAMRA-FP serine hydrolase probe (Thermo Scientific) was added to each sample and incubated for 30 minutes. The reactions were quenched with addition of SDS loading dye and boiling for 10 minutes. To quantitate the amount of active protein, a standard of the probe alone was prepared by serial dilution with ABPP buffer. All samples were resolved using SDS-PAGE and imaged using a Typhoon Trio+ imager (GE Healthcare Life Sciences). ImageQuant TL software was used for quantification of bands corresponding to the TAMRA-FP serine hydrolase probe alone, probe-labeled LipY and probe-labeled LipYΔPE.

### Enzymatic Assays

Michaelis-Menten kinetics were measured as previously described[24]. Briefly, LipY or LipYΔPE was diluted in assay buffer (20 mM Tris pH 8, 150 mM NaCl, 0.2% Fatty acid free BSA, and 0.4% (v/v) Triton X-100) to a set concentration according to quantification by ABPP. Next, the fluorogenic substrate 1,2-Di-O-lauryl-rac-glycero-3-(glutaric acid 6-methylresorufin ester) known as DGGR (Sigma Aldrich) was added. Substrate hydrolysis was measured by monitoring fluorescence with a 529 nm excitation wavelength and reading emission at 600 nm with a 590 nm cutoff filter over 30 minutes at 37°C. Non-enzymatic hydrolysis was subtracted and initial rates of hydrolysis were calculated using the first 10% of data. Initial rates were plotted against substrate concentration and fitted to the Michaelis-Menten equation using KaleidaGraph software.

### Circular Dichroism Measurements

Thermal stability assays were measured as previously described with some modifications[25]. Briefly, LipY or LipYΔPE was diluted to 10 μM in Buffer D and data were collected at 20°C. Molar ellipticity of thermal denaturation was monitored at 222 nm from 4–90°C. Buffer D was used as a blank and all data were converted to mean residue ellipticity (MRE) using Eq 1, where Θ is MRE, signal is the CD signal, *C* is the protein concentration (mM), *n* is the number of amino acids and *l* is the cell path length (cm)[26].

Θ=(100×signal)÷(C×n×l) (1)

## Results

### Detergent separates LipY into 2 distinct peaks

**Fig 1:**
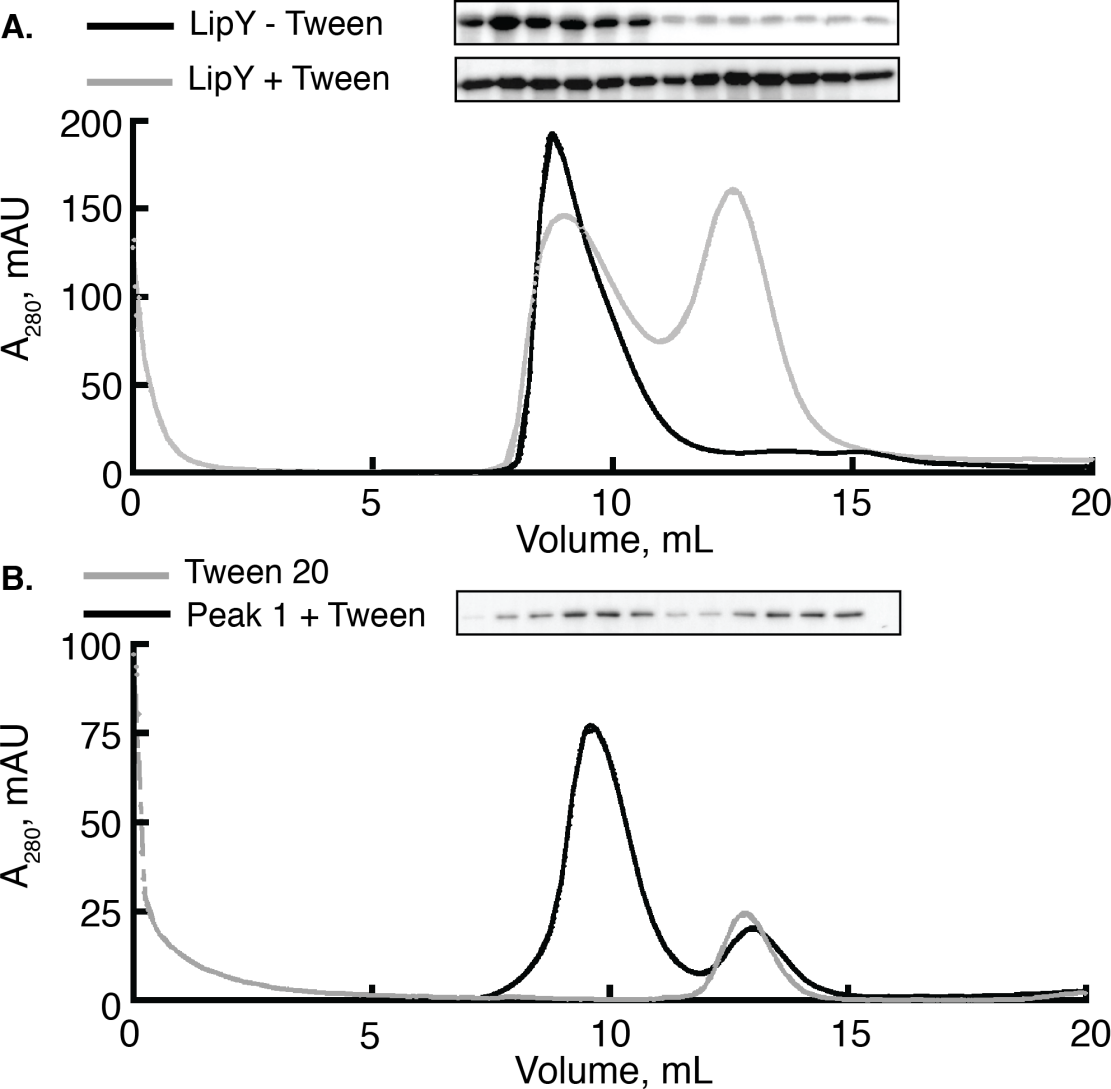
Size exclusion chromatography of LipY with or without Tween 20. (A) LipY purified via Nickel affinity chromatography was resolved on a Superdex 200 gel filtration column with (gray trace) or without (black trace) pre-incubation with a 270 fold molar excess of Tween 20. In the presence of detergent a second peak in the included volume appears whereas a single peak in the included volume is observed when no detergent is present. (B) LipY purified in the void volume (Peak 1) was pre-incubated with a 270 fold molar excess of Tween 20 and subjected to size exclusion chromatography (black trace). This trace is overlayed with a trace of 6 mM Tween 20 alone (gray trace). Western blots against the His tag of LipY were used to detect the presence of protein eluted from the gel filtration column in each experiment.

Most PE/PPE proteins do not have well-characterized functions, but LipY is a well-characterized lipase. We therefore used LipY to determine how the PE domain affects its enzymatic activity. To carry out these studies we first needed to resolve the oligomeric state of active LipY. Although LipY is a 45 kDa protein, previous reports of its purification show that it elutes from a gel filtration column at or near the void volume, indicating a protein of over 600 kDa[13]. LipY purified from this void fraction exhibited TG hydrolysis activity and in a previous study was used for its biochemical characterization[13]. We developed an improved purification protocol in which LipY migrates in the included volume of a Superdex 200 gel filtration column (Fig 1A, gray trace). To achieve this, we utilized detergent micelles to help solubilize nickel-purified LipY. Lipases function at the lipid-water interface, and they are frequently purified and crystallized in the presence of detergent micelles[27, 28]. After addition of Tween 20 (CMC = 0.6 mM) at a 270-fold molar excess over LipY, we observed that LipY elutes from a size exclusion column in 2 distinct peaks: one is in the void volume (Peak 1) and one in the included volume (Peak 2). By contrast, when LipY was not supplemented with detergent, only Peak 1 was observed (Fig 1A, black trace). We next tested the contents of both peaks by Western blot. These experiments confirmed that LipY migrates mainly in Peak 1 in the absence of Tween 20 but migrates in Peak 2 when Tween 20 is added (Fig 1, inset).

This result raised the question of whether LipY was migrating independently or in/on the detergent micelles. We next ran a sample containing only Tween 20 micelles on the size exclusion column (Fig 1B). This trace revealed that LipY from Peak 2 matched the elution profile of the Tween 20 micelles. Next, we wanted to determine if the protein in Peak 1 was refractory to solubilization by detergent. We supplemented LipY isolated from Peak 1 with Tween 20, repeated the purification strategy using gel filtration and observed both Peak 1 and Peak 2 in the elution profile (Fig 1B, black trace). The appearance of LipY in both peaks was confirmed via immunoblot analysis. Taken together, these results indicate that Tween 20 micelles can extract LipY from a large, soluble aggregate.

### The PE Domain of LipY contributes to formation of LipY aggregates in Peak 1

**Fig 2:**
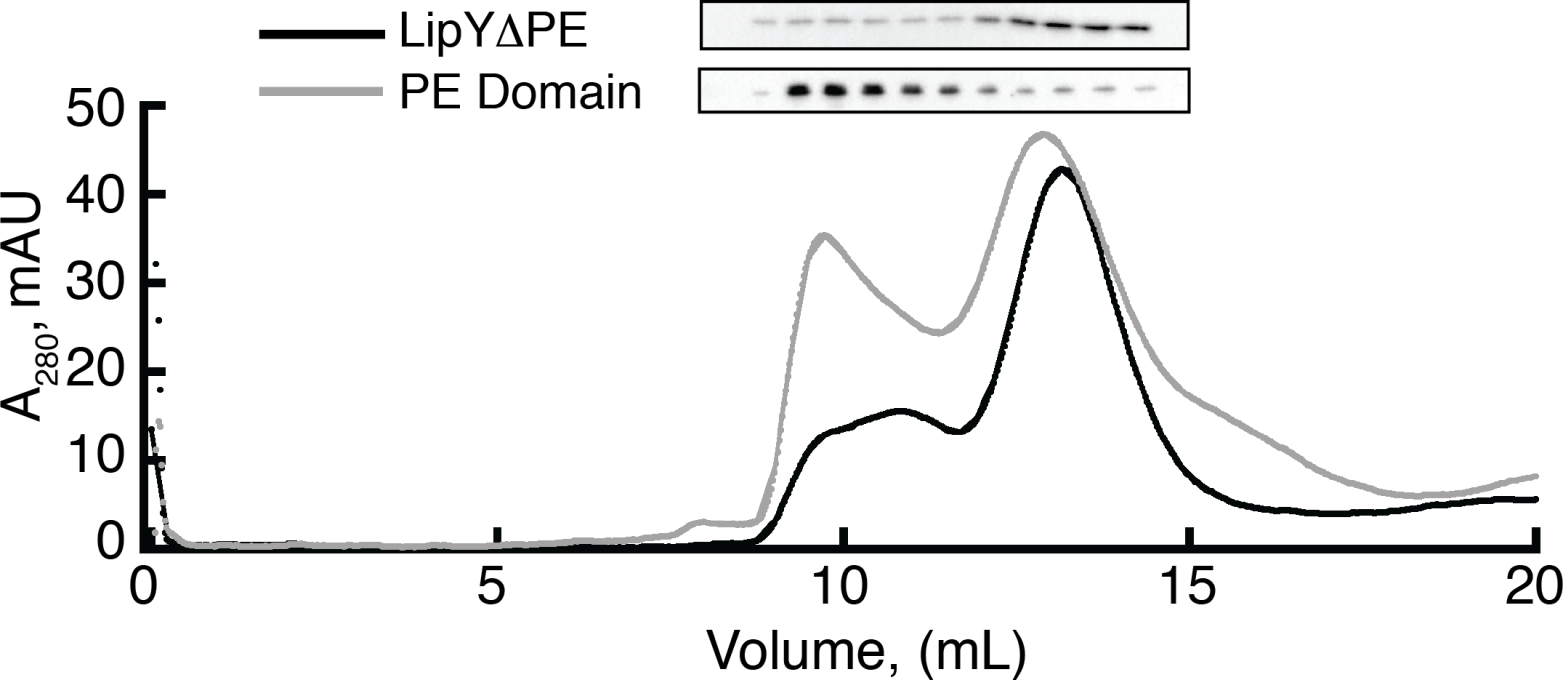
The PE domain contributes to LipY aggregation *in vitro*. LipYΔPE and the PE domain were resolved on a Superdex 200 gel filtration column. Both proteins were pre-incubated with a 270 fold molar excess of Tween 20 for 1 hour prior to being resolved on the column. In the gray PE domain trace, Peak 1 (~9.5 mL) elutes near the void volume and Peak 2 (~13 mL) elutes in the included volume. In the black LipYΔPE trace, a weak A_280_ signal was observed between 9 and 12 mL and a single distinct peak (Peak 2) elutes in the included volume. Western blots against the His tag of each construct show that the majority of the PE domain elutes in the void volume while the majority of LipYΔPE elutes in the included volume.

We next sought to determine whether the PE domain of LipY or the mature lipase (LipYΔPE) is responsible for LipY aggregation. On one hand, PE proteins are known to form dimers with their cognate PPE partner for recognition and exportation by unique type VII secretion systems[8, 29]. Conversely, there are many examples of bacterial lipases that are prone to aggregation upon purification[28, 30]. To answer this question, we first expressed and purified recombinant LipYΔPE (residues 150–437) and recombinant PE domain (residues 1–149) using nickel affinity chromatography. Consistent with the purification of LipY, we added Tween 20 at a 270 fold molar excess over protein to help solubilize the purified proteins prior to size exclusion chromotography. The elution profile for the PE domain revealed the formation of two distinct peaks as we previously observed with full length LipY (Fig 2). However, the elution profile of LipYΔPE featured a distinct peak in the included volume with a smaller, broad peak in the void volume. Immunoblot analysis of the size exclusion column fractions of LipYΔPE and the PE domain revealed that the majority of purified PE domain is in the void volume (Peak 1) whereas the majority of purified LipYΔPE is in the included volume (Peak 2). The data show that removal of the PE domain from purified LipY dramatically reduces its aggregation into Peak 1. Taken together with data from Fig 1, these results demonstrate that the PE domain drives the aggregation of LipY.

### LipY from Peak 2 is more active than LipY from Peak 1

**Fig 3:**
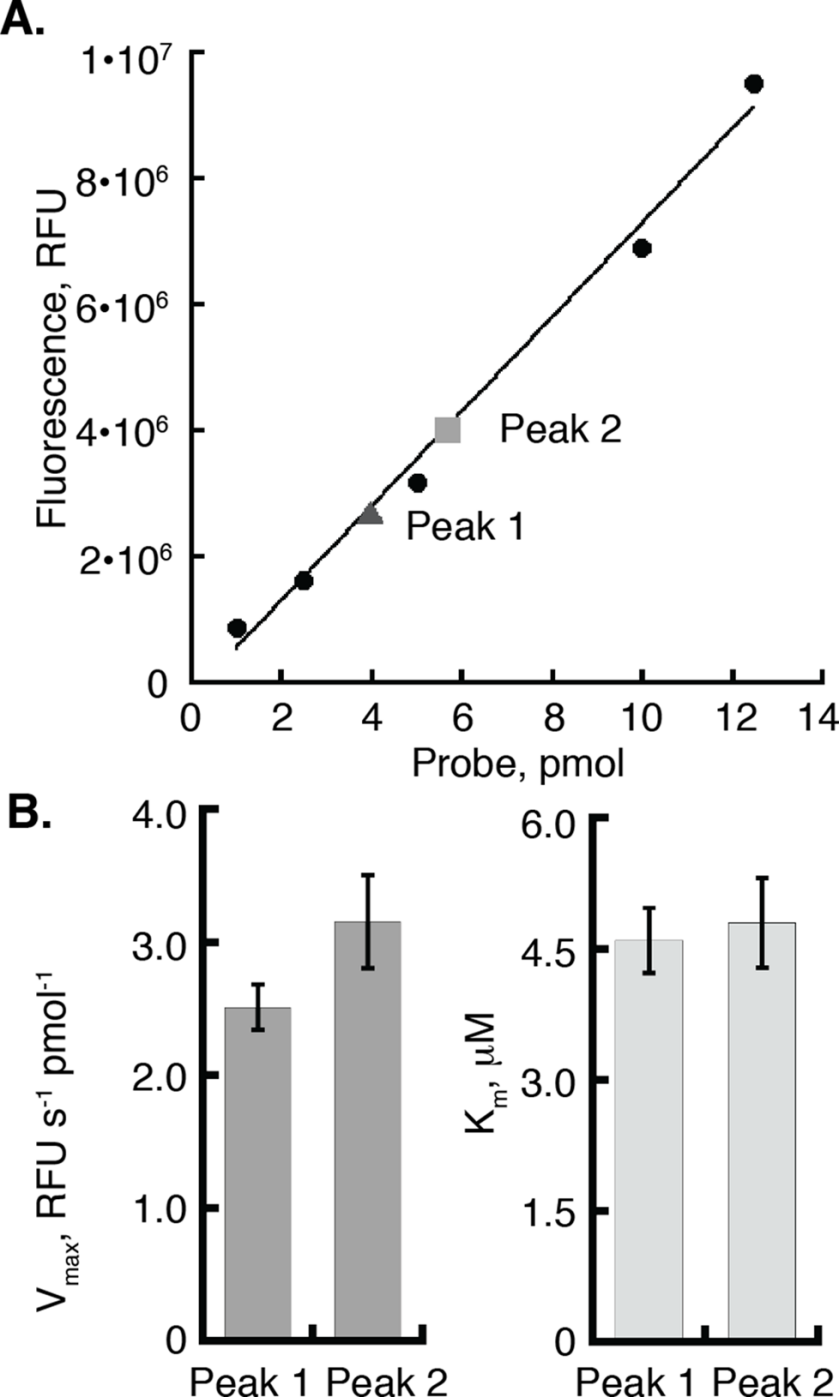
There are more active LipY molecules in peak 1 compared to peak 2. (A) Equal concentrations of *total* LipY Peak 1 and Peak 2 were incubated with a 1.5 molar excess of TAMRA-FP serine hydrolase probe for 30 minutes at room temperature. A standard was created using the probe alone. The amount of active Peak 1 and Peak 2 were calculated based on the standard curve. (B) Bar graph showing the V_max_ and K_m_ from Michaelis-Menten curves comparing equal amounts of *total* LipY from Peak 1 and Peak 2 using the DGGR substrate. Error bars represent the standard error of the mean of 6 independent measurements[32].

Although LipY from Peak 1 elutes in the void as a high molecular weight aggregate, our lab and others have observed that the LipY purified from Peak 1 maintains lipase activity[13]. We therefore set out to compare the fraction of active LipY purified in Peak 1 and Peak 2. First, we used activity-based protein profiling (ABPP) to measure the amount of active enzyme in both Peak 1 and Peak 2. In this method, the reactive fluorophosphonate group of the activity-based probe (ABP) forms a covalent bond with the active site serine residue of *active* serine hydrolases, including lipases with a 1:1 ratio of ABP:active site[31]. We calculated the amount of active enzyme in each peak (plotted in Fig 3A). LipY in Peak 2 contains more active enzymes (~45% active) than Peak 1 (~32% active). We then compared the Michaelis-Menten kinetics of LipY from Peak 1 and Peak 2 using the long-chain lipase substrate DGGR (Fig 3B). These experiments confirmed that Peak 2 (V_max_ = 3.2 ± 0.3 RFU s^-1^ pmol^-1^ *total* LipY) is more active as compared to Peak 1 (V_max_ = 2.5 ± 0.2 RFU s^-1^ pmol^-1^ *total* LipY). The K_m_ values for Peak 1 and Peak 2 are within the experimental error of each other (5.0 ± 1.5 and 4.8 ± 1.1 μM, respectively).

To confirm that Peak 2 is more active than Peak 1 we used an established method to compare the activities of the two peaks[33]. We used a separate protein purification as we observed that the exact fraction of active protein in each peak varied from purification to purification but Peak 2 was always more active than Peak 1. Tetrahydrolipstatin (THL) is a lipase inhibitor that covalently modifies the active site of lipases[34, 35]. LipY (5 nM) was titrated with increasing concentrations of THL, and the residual lipase activity was compared to untreated LipY. Residual LipY activity was measured using the small, soluble substrate *p*-Nitrophenyl butyrate (pNPB) at a concentration of 200 μM, which is 2 fold over the K_m_ for LipY hydrolysis of pNPB. Consistent with data shown in Fig 3, LipY in Peak 2 is more active than LipY in Peak 1 (S3 Fig). Protein purified from Peak 2 was used in all subsequent experiments as our studies show that it is the disaggregated, more active form of the enzyme.

### LipYΔPE is more active than LipY

**Fig 4:**
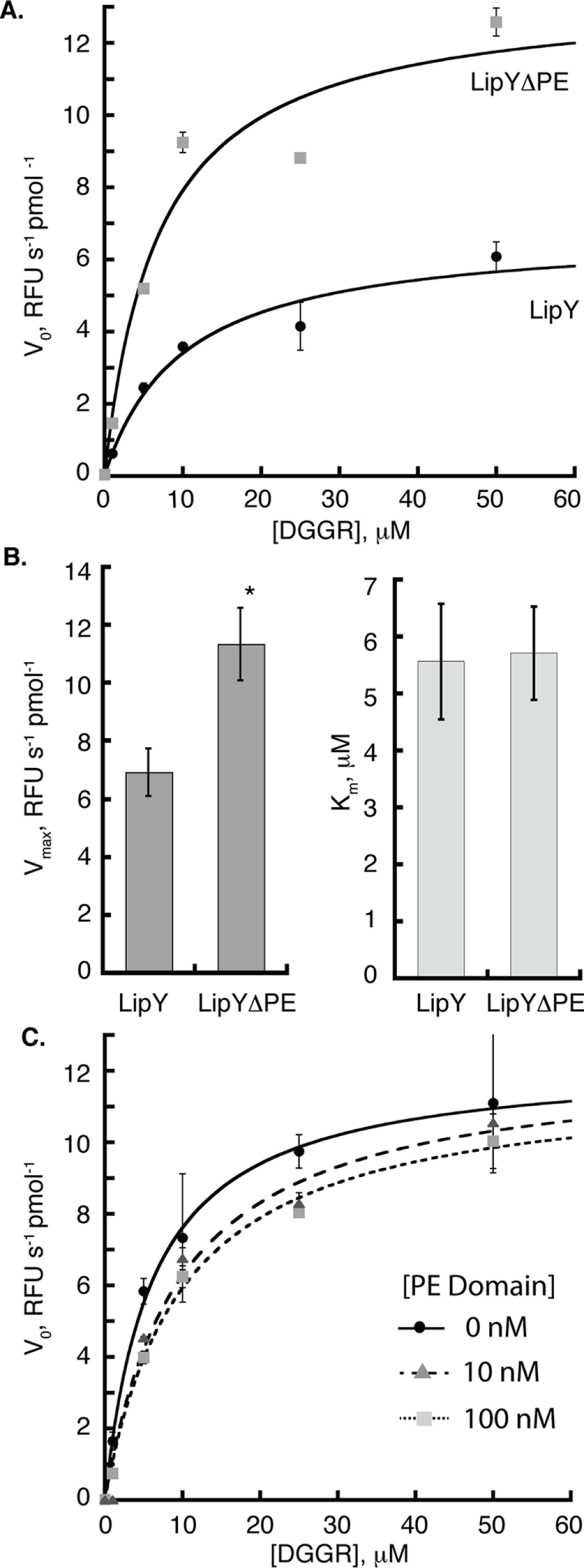
LipY’s PE domain downregulates its enzymatic activity. (A) Equal amounts of *active* LipY and LipYΔPE as determined by ABPP were used to compare the Michaelis-Menten kinetics of the two constructs. Hydrolysis of the lipase substrate, DGGR was monitored at 37°C. Initial velocities for a representative data set were fit to the Michaelis-Menten equation and plotted against substrate concentration. (B) Analysis of multiple data sets reveals that LipYΔPE has a V_max_ that is approximately two fold higher than the V_max_ for LipY (left graph). However, the K_m_ is not affected (right graph). Error bars represent the standard error of the mean of 6 independent measurements[32]. “*” corresponds to p<0.015 of the V_max_ of LipY compared to LipYΔPE. (C) LipYΔPE was titrated with 0, 10 or 100 nM PE domain and incubated for 10 minutes before testing its residual lipase activity as described in (A).

A study comparing cells expressing LipY vs. LipYΔPE reported that the crude cell wall fraction from the LipYΔPE-expressing cells had greater enzymatic activity[23]. These results suggest that the PE domain may down-regulate LipY enzymatic activity. However, because this result was obtained using a crude cell wall fraction, it could also be explained by loss of an inhibitor-binding site as a result of the deletion of the PE domain. Additionally, the authors created the initial LipYΔPE construct by deleting the first 99 residues from LipY’s N-terminus. Subsequently, experimental evidence showing the precise cleavage site (after glycine 149) for removal of the PE domain emerged[19]. We tested the role of the PE domain in LipY activity by comparing full length LipY and LipYΔPE generated using the physiological cleavage site. To compare the activities of LipY and LipYΔPE, we first employed the method used in Fig 3A to calculate the amount of active protein. After determining the amounts of active LipY and LipYΔPE using the ABPP method, we compared Michaelis-Menten kinetics of equal amounts of active protein using DGGR as a substrate. These data show that LipYΔPE is more active than LipY, as shown by the representative plot in Fig 4A. Analysis of multiple data sets revealed V_max_ values of 6.3 ± 0.8 and 11.3 ± 1.3 RFU s^-1^ pmol^-1^ for LipY and LipYΔPE, respectively, indicating a significant difference in activity (p<0.015) between the two LipY variants (Fig 4B, left graph). Loss of the PE domain does not affect substrate binding, as the K_m_ values are equivalent for LipY and LipYΔPE (Fig 4B, right graph).

As shown in Fig 2, we successfully expressed and purified the 149-amino acid PE domain of LipY. We tested the ability of the purified PE domain to act as an inhibitor of LipYΔPE *in trans*. Increasing amounts of PE domain were incubated with a constant amount of LipYΔPE for 10 minutes prior to testing lipase activity. The initial velocity was plotted against substrate concentration and data for each concentration of PE domain tested were fitted to the Michaelis-Menten equation. We observed negligible differences in activity of LipYΔPE alone and LipYΔPE pre-incubated with PE domain (Fig 4C). This lack of inhibition indicates that the PE domain only inhibits LipY when it is part of the same molecule.

### The PE domain does not affect thermal stability of LipY

**Fig 5:**
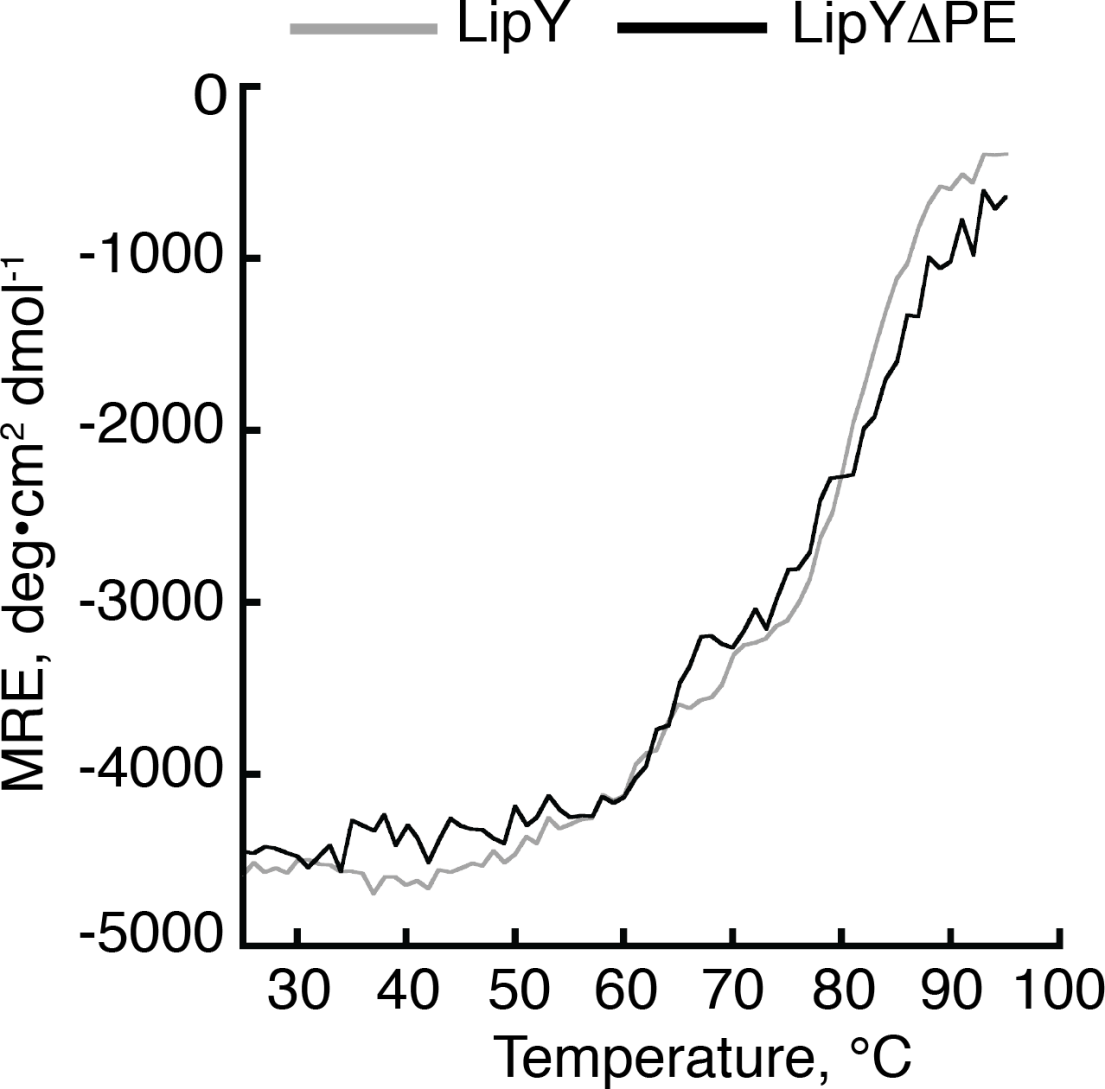
LipY and LipYΔPE share similar thermal unfolding profiles. CD temperature scans of LipY (black) and LipYΔPE (gray) monitored at 222 nm between 25 and 95°C. Data points were collected every 1°C.

Finally, we asked if the PE domain might affect the thermal stability of full length LipY. We probed the thermal stability of both LipY and LipYΔPE by measuring their thermal denaturation using circular dichroism (CD) spectroscopy. Based on far-UV CD scans of both proteins, we elected to measure unfolding at 222 nm. Both constructs showed a single thermal unfolding transition. Furthermore, the melting temperatures (T_m_) of the two constructs were very similar (Fig 5). Both constructs had T_m_s near 75°C, which suggests that even in the absence of the PE domain LipY is highly stable. This is in good agreement with a previous study measuring the activity of LipY as a function of temperature. In this study LipY retained partial activity even after incubation at 60°C for 15 minutes prior to an activity assay[13].

## Discussion

PE and PPE proteins of *Mtb* were first described almost 17 years ago, and their molecular functions are still not fully understood. Studies with individual PE/PPE proteins suggest that they serve to target their C-terminal cargo to the type VII secretion systems[19, 36, 37]. However, the physiological utility of employing a large, aggregation prone domain to target proteins for secretion is not clear, and it is tempting to speculate that the PE domain may have additional roles. LipY represents one of the best candidates for studying the PE domain because its enzymatic activity is well established and the cleavage site for the PE domain is known. In the case of LipY, the PE domain has at least two distinct functions. First, it is clear that the YxxxD/E motif acts as a secretion signal for export of the mature lipase to the cell wall[20]. Second, the PE domain has been implicated as a regulator of enzyme activity[23]. Here, we utilize biochemical assays to investigate this observation because experiments initially describing this regulatory function were carried out using crude cell wall fraction. Thus, it is possible that other factors interacted with the PE domain to regulate LipY activity. Furthermore, only the first 99 amino acids were removed from LipY, as it was only later demonstrated that the PE domain precedes a linker region and the physiological cleavage site is after amino acid position 149[19].

We therefore compared the lipase activity of purified LipY and LipYΔPE generated using the physiological cleavage site. We first needed to de-aggregate LipY and did so by utilizing Tween 20 detergent micelles. These experiments led to the finding that the PE domain of LipY provides an interface for its aggregation, as the isolated PE domain aggregated whereas LipYΔPE was less susceptible to aggregation. Our data imply that PE proteins, especially those associated with the cell wall of *Mtb*, are prone to aggregation via their PE domain. This should be taken into consideration when purifying proteins from this family. We next quantified the amount of active enzyme molecules using an activity-based method to compare equal amounts of active LipYΔPE and LipY. We observed a clear increase in the maximum reaction velocity of purified LipYΔPE compared to LipY thus confirming that the PE domain does indeed down-regulate LipY activity. Finally, we added the isolated PE domain back to LipYΔPE and found that it did not inhibit *in trans*.

Thus, the PE domain must be attached to the mature lipase in order to inhibit lipase activity. It is likely that this connection is necessary because it increases the local concentration of the PE domain at the site of inhibition. In data not shown, we tested PE domain concentrations up to 0.5 μM but were not able to test higher concentrations due to the risk of PE domain aggregation. Our kinetic analysis shows that LipY has a lower V_max_ and an equivalent K_m_ as compared to LipYΔPE. This is characteristic of noncompetitive inhibition, in which an inhibitor affects enzyme activity by binding to a site other than the substrate-binding site (for example an allosteric site)[38]. However, understanding the precise molecular mechanism by which the PE domain reduces LipY activity will require further mechanistic studies.

Our study includes a useful technical advance. We used three different methods to compare the activities of LipY from Peak 1 and Peak 2: ABPP, active site titration using THL, and Michaelis-Menten kinetics using a model triglyceride substrate. All methods demonstrated that LipY from Peak 2 was more active than LipY from Peak 1. Generally, ABPP has been used as a proteomics tool for screening inhibitors and identifying enzymes of interest in cell fractions[39]. Our study shows that the TAMRA-FP serine hydrolase activity based probe is also useful to quantify enzyme active sites. Because the ABPs only label active protein, this approach offers a quicker, easier way to quantitate enzyme active sites than using pre-steady state kinetics or active site titration.

LipY functions both intracellularly and extracellularly, and the PE domain is removed upon secretion[19]. Our data suggest that the absence of the PE domain allows the mature lipase to better hydrolyze TGs outside the cell, thus tuning LipY activity to its environment. The observations that the PE domain reduces LipY activity leads to the question of whether this is a LipY-specific phenomenon or a common occurrence. Currently, few other PE/PPE proteins have known enzymatic functions. It is also not well established if other PE/PPE domains are cleaved upon secretion. However, some studies do hint that this may be true for certain PE/PPE proteins. For example, a 2-D gel analysis showed that a number of proteins dependent on ESX-5 for secretion into the culture were present at lower than expected molecular weights[11]. Several PE/PPE proteins were among this group. As the biological functions of additional PE/PPE proteins are uncovered, careful biochemical analysis will provide a better understanding of the full significance and functionality of PE/PPE domains.

## Supporting Information

S1 Fig LipY and LipYΔPE after purification. (A) 50 pmoles each of LipY and LipYΔPE were loaded on a 12% SDS-PAGE gel, which was stained with Sypro Orange and imaged using a BioRad ChemiDoc Imaging System. The purity of the protein is demonstrated in the lane profiles to the right, which were analyzed using Image Lab 4.1.
(TIF)

S2 Fig LipY and LipYΔPE are active on a natural triglyceride substrate. LipY and LipYΔPE from Peak 2 (50 nM active protein) were incubated with triglyceride-rich lipoprotein particles. Free fatty acids were released upon substrate hydrolysis; these were measured using a colorimetric assay as described in S1 File. Three independent measurements were taken and error bars represent the standard deviation.
(TIF)

S3 Fig Active site titration and kinetics of LipY in peak 1 and peak 2. (A) Titration curve of LipY Peak 1 and Peak 2 inhibited with THL. Residual enzyme activity was measured using *p*-Nitrophenyl butyrate and normalized to uninhibited LipY. Activity versus ratio of THL to enzyme was plotted and the linear portions of each set of data were fit linearly. (B) Michaelis-Menten curves comparing equal amounts of *total* LipY from Peak 1 and Peak 2 using the DGGR substrate.
(TIF)

S1 File Supplemental Methods and References. (DOCX)
