# Supplementary figures and images for: Modulation of the Activity of Mycobacterium tuberculosis LipY by Its PE Domain
Source: PLoS One. 2015 Aug 13;10(8):e0135447. doi: 10.1371/journal.pone.0135447 (PMC4536007; doi:10.1371/journal.pone.0135447)

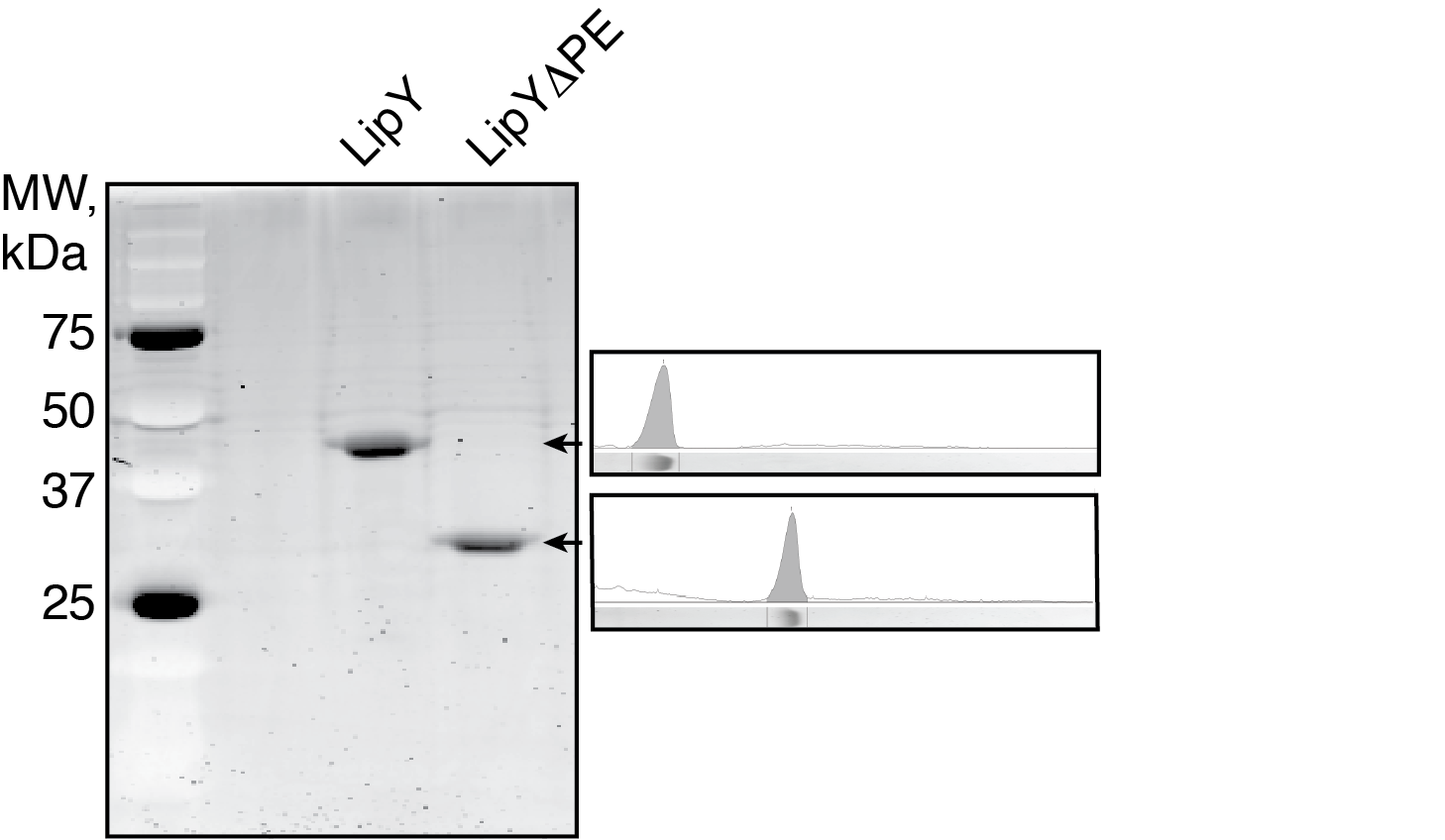

Supplement: S1 Fig — (A) 50 pmoles each of LipY and LipYΔPE were loaded on a 12% SDS-PAGE gel, which was stained with Sypro Orange and imaged using a BioRad ChemiDoc Imaging System. The purity of the protein is demonstrated in the lane profiles to the right, which were analyzed using Image Lab 4.1. (TIF) [file pone.0135447.s001.tif]

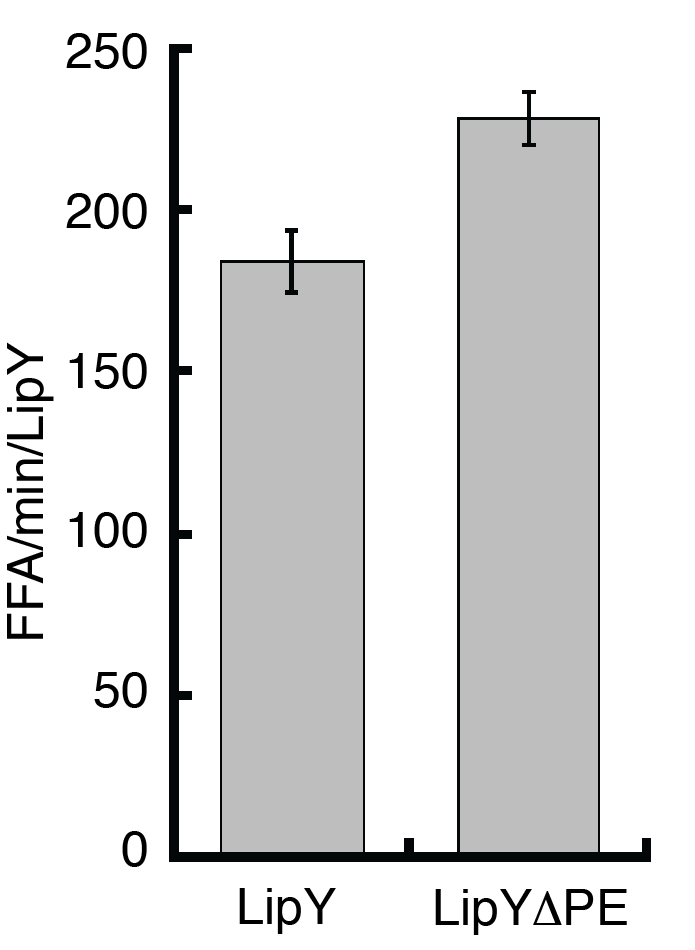

Supplement: S2 Fig — LipY and LipYΔPE from Peak 2 (50 nM active protein) were incubated with triglyceride-rich lipoprotein particles. Free fatty acids were released upon substrate hydrolysis; these were measured using a colorimetric assay as described in S1 File. Three independent measurements were taken and error bars represent the standard deviation. (TIF) [file pone.0135447.s002.tif]

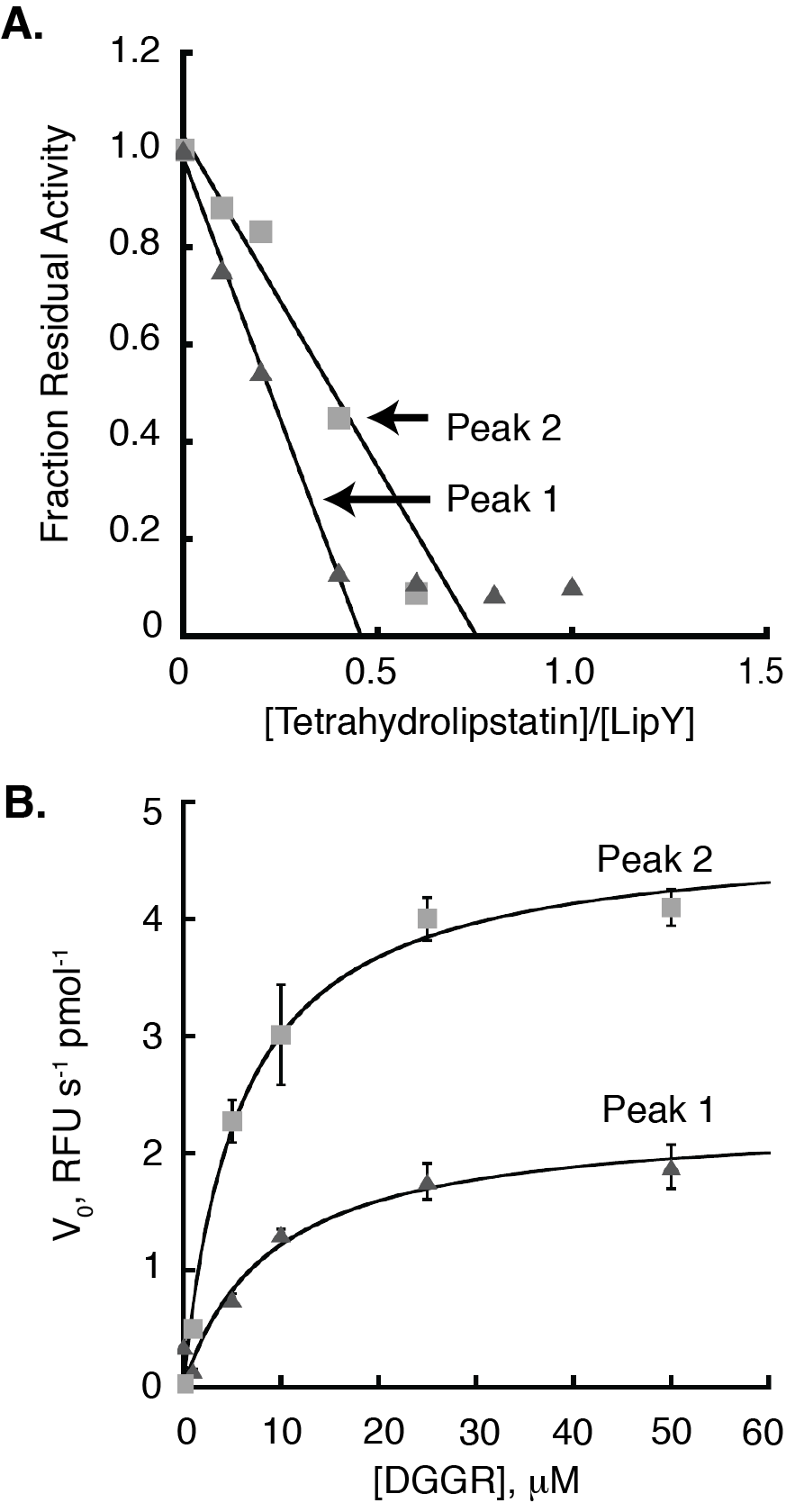

Supplement: S3 Fig — (A) Titration curve of LipY Peak 1 and Peak 2 inhibited with THL. Residual enzyme activity was measured using p-Nitrophenyl butyrate and normalized to uninhibited LipY. Activity versus ratio of THL to enzyme was plotted and the linear portions of each set of data were fit linearly. (B) Michaelis-Menten curves comparing equal amounts of total LipY from Peak 1 and Peak 2 using the DGGR substrate. (TIF) [file pone.0135447.s003.tif]
