# Supplementary material for: Modulation of the Activity of Mycobacterium tuberculosis LipY by Its PE Domain
Source: PLoS One. 2015 Aug 13;10(8):e0135447. doi: 10.1371/journal.pone.0135447 (PMC4536007; doi:10.1371/journal.pone.0135447)
Supplement: S1 File — (DOCX) [file pone.0135447.s004.docx]

**Supplemental Methods**

**Isolation of Plasma Derived Triglycerides**

Plasma derived triglyceride rich lipoprotein (TRL) particles were isolated as previously described[1].

**Quantification of Substrate Triglyceride Levels**

The triglyceride content of the sample was quantified as previously described with some modifications[2]. Briefly, samples were diluted in PBS and incubated with 7.25 μM Candida Rugosa Lipase (Sigma Aldrich) in hydrolysis buffer (133 mM KPO_4_ and 0.25% FFA BSA) for 30 minutes at 37 °C. Next, quantification buffer (133 mM KPO4, 3.3 mM McCl2, 4.4mM ATP, 0.6 mM TOOS, 6 U/mL POD, 1.73 mM bromophenol, 1.73 mM aminoantopyrine, 0.1 mM FAD, 0.5% Triton X-100, 0.25 U/mL Glycerol Kinase (Fisher Scientific), 4 U/mL Glycerolphosphate oxidase (Fisher Scientific)) was added each sample and allowed to incubate for 15 minutes at 37 °C. A sample containing no lipase was used for background subtraction for the hydrolyzed sample. Samples were read on a Spectromax M5 plate reader at 555 nm. Samples were fit to the standard curve to give the final triglyceride concentration.

**Triglyceride Hydrolysis Assays**

Reaction samples were loaded into wells of a clear 96-well plate (Greiner). 50 nM LipY was incubated with TRLs (at a concentration of approximately 2.6 mM triglycerides). Individual reactions were quenched by the addition of Orlistat (Cayman Chemical Company, Ann Arbor, Michigan, USA) to a final concentration of 80 μM. Released free fatty acids (FFA) were quantified using a slight adaptation of a previously reported assay[3]. Briefly, reagent A (final concentrations: 133 mM KPO_4_ pH 7.5, 3.3 mM MgCl_2_, 4.4 mM ATP, 1 mM CoA, 0.055 U/mL ACS, 0.5% Triton X-100) was added to each sample (including palmitate standards) and the mixtures were incubated for 15 minutes at 37°C. Reagent B (final concentrations: 133 mM KPO_4_ pH 7.5, 0.6mM TOOS, 6 U/mL Horse POD, 1.73 mM dibromophenol, 1.73 mM 4-aminoantipyrine, 0.1 mM FAD, 10 mM NEM, 5 U/mL ACO, 0.5% Triton X-100) was then added to each sample and further incubated at 37°C for 10 minutes. Samples were assayed in triplicate. Samples were read on a Spectromax M5 plate reader at 555 nm. FFA release for each sample was quantified using a standard curve of palmitate.

**References For Supporting Information**

1. Chung BH, Wilkinson T, Geer JC, Segrest JP. Preparative and quantitative isolation of plasma lipoproteins: rapid, single discontinuous density gradient ultracentrifugation in a vertical rotor. J Lipid Res. 1980;21(3):284-91. Epub 1980/03/01. PubMed PMID: 7381323.

2. McGowan MW, Artiss JD, Strandbergh DR, Zak B. A peroxidase-coupled method for the colorimetric determination of serum triglycerides. Clin Chem. 1983;29(3):538-42. Epub 1983/03/01. PubMed PMID: 6825269.

3. Okabe H, Uji Y, Nagashima K, Noma A. Enzymic determination of free fatty acids in serum. Clin Chem. 1980;26(11):1540-3. Epub 1980/10/01. PubMed PMID: 6774837.
